# Comparison of the antimicrobial activity of propolis extracts obtained by means of various extraction methods

**DOI:** 10.1007/s13197-019-04009-9

**Published:** 2019-09-30

**Authors:** Katarzyna Pobiega, Karolina Kraśniewska, Dorota Derewiaka, Małgorzata Gniewosz

**Affiliations:** Department of Biotechnology, Microbiology and Food Evaluation, Faculty of Food Sciences, Warsaw University of Life Sciences – SGGW, Nowoursynowska 159c str., 02-776 Warsaw, Poland

**Keywords:** Propolis, Extraction method, Sonication, Antimicrobial activity, GC–MS chemical composition

## Abstract

The objective of the study was to compare the antimicrobial activities of ethanolic propolis extracts obtained using different extraction methods. Extraction of propolis was carried out using 70% ethanol, propolis to ethanol ratios of 1:10 and 1:5, extraction times of 1 or 7 days, and shaking extraction (SE), ultrasound-assisted extraction (UAE), and ultrasound-assisted shaking extraction (SUAE) methods. A total of 12 propolis extract lyophilizates were obtained. Samples were tested for extraction yield and for total phenol content by the Folin–Ciocalteau colourimetric method, and total flavonoid content using a spectrophotometric method. GLC/MS was used for the identification of chemical compounds in selected extract lyophilizates. Antimicrobial activity against selected bacterial and fungal species was assessed using the disk diffusion method. Propolis extracts obtained as the result of 1-day and 7-day shaking extraction followed by 20 min of ultrasound-assisted extraction (SUAE) had better antimicrobial properties as compared to those obtained by SE or UAE alone. SE and UAE gave lower extraction yields as well as lower phenol and flavonoid contents compared to SUAE. No differences were observed with regard to the qualitative composition of extracts obtained by any of the methods. It is best to obtain the extract using the combined method of 1-day extraction and 20-min sonication.

## Introduction

Propolis is produced by bees from tarry and balsamic substances found within flower buds or the bark of deciduous trees, as well as from resins exuding from damaged parts of trees. All these substances are then modified by the addition of wax and apian gland secretions (Bankova et al. 2016b). Propolis is a sticky, resin-like substance of a tan, dark-yellow, orange, brown, or even green color and a very distinctive, intense scent. The composition of propolis varies depending on the region, climate, and prevalent floral species. In moderate climates, various species of poplar and alder trees are used for its production. The resin content (flavonoids and related phenolic acids) of European propolis accounts for about 50% of its composition. Other ingredients include beeswax (30%), aromatic and oily substances (10%), as well as pollen and mechanical admixtures (5% each) (Burdock 1998; Bankova et al. 2002). Studies on the chemical composition of propolis have been conducted for many years. Nearly 420 chemical substances have been identified to date in propolis samples originating from different geographical regions of the world. The main constituents include flavones, flavonols, flavanone, and dihydroflavonoids, as well as phenylpropanoid derivatives (Milojković-Opsenica et al. 2016). Thanks to the presence of these substances, propolis is characterized by a broad range of biological activities. Propolis has been documented to possess antibacterial, antifungal, antiviral, antiparasitic, antioxidative, anticancer, anti-inflammatory, antiulcer, and antidiabetic effects (Pasupuleti et al. 2017; Al-Ani et al. 2018).

Raw propolis is not suitable for food technology, pharmaceutical or cosmetic industry applications due to the high content of impurities which have to be removed (Galeotti et al. 2018). To this end, bioactive constituents of propolis are extracted using organic solvents (Gómez-Caravaca et al. 2006). The process is expected to eliminate inert materials while preserving the polyphenolic fractions (Gómez-Caravaca et al. 2006). Propolis extracts for use in food production are usually obtained using ethanolic solutions or water (Kubiliene et al. 2015; Bankova et al. 2016a). Extraction with ethanol is particularly suitable for obtaining deparaffinated extracts rich in polyphenolic components. On the other hand, extraction with pure water is suitable for obtaining extracts containing water-soluble phenolic acids (Gómez-Caravaca et al. 2006).

With regard to the techniques for the extraction of raw propolis, room temperature maceration and hot reflux extraction (HRE) were widely used in the past (Trusheva et al. 2007). More advanced techniques used in propolis extraction include microwave-assisted extraction (MAE) (Pellati et al. 2013; Hamzah and Leo 2015), ultrasound-assisted extraction (UAE) (Yeo et al. 2015), and supercritical carbon dioxide extraction (De Zordi et al. 2014). Important advantages of these methods include shorter extraction times, higher extraction yields, and lower solvent consumption compared to conventional methods (Zhou et al. 2009; Zhang et al. 2011).

UAE uses ultrasonic energy (> 20 kHz) for extraction using either an ultrasonic bath and/or an ultrasonic probe. It works on the principle of making cavitation bubbles which collapse and produce higher shear, which results in complete extraction (Belwal et al. 2018). Ultrasound accelerates the mixing of the components and facilitate contact between the extracted material and fresh solvent, as well as continuous removal of the stagnant layer barrier. In addition, ultrasound contributes to fragmentation of the extracted material and thus to the enhancement of its exposure to the solvent. It also enlarges the cell pores so that the cells are penetrated by the solvent faster. All the above processes result in accelerated mass exchange between the material and the solvent, resulting in increased extraction yields (Vinatoru et al. 2017).

Harvesting, transporting and packaging are the main sources of microbial contamination. Plant raw materials are carriers of many pathogenic bacteria, such as *Escherichia coli* and *Staphylococcus aureus*. In turn, during storage they are exposed to spoilage, which is responsible for the development of fungi such as: *Mucor mucedo*, *Alternaria solani*, *Colletotrichum gloeosporioides*. Improper cleaning or processing of such products before consumption can lead to epidemics. For this reason, all the time looking for natural substances, which on the one hand will protect vegetable raw materials against the development of pathogens and on the other hand against the spoilage caused by fungi (Curifuta et al. 2012; Gniewosz et al. 2014; Kraśniewska et al. 2015; Oni et al. 2018).

The objective of the study was to compare the antimicrobial activities of ethanolic propolis extracts obtained using three different extraction methods. The analysis focused on the effect of the propolis extraction mixture to ethanol weight ratio, time of extraction, as well as shaking and ultrasound-assisted extraction status on the antibacterial efficacy of the obtained propolis extracts. The results were correlated with the determined active substance content for a more rational selection of an optimum procedure to obtain antimicrobial propolis extracts.

## Materials and methods

### Materials

Raw propolis was collected in 2017 from an apiary in Bałtów (southern part of central Poland, 21°32′E; 51°01′N). The material was loose, dark brown in colour, and had a characteristic scent. Prior to the analysis propolis samples were kept at room temperature in the dark.

### Preparations of dry ethanolic extracts of propolis (EEP)

Two mixtures were prepared as follows: 10 g of pulverized sample was weighed and dissolved in 100 mL of 70% ethanolic solution in a 1:10 (w/v) ratio, while another 10 g of pulverized sample was weighed and dissolved in 50 mL of 70% ethanolic solution in a 1:5 (w/v) ratio. Next, samples were extracted using three different extraction methods. In the first method (SE), samples were shaken (200 rpm) at 28 °C for 1 or 7 days (SM-30 Control, Edmund Bühler, Germany). In the second method (UAE), samples were subjected to ultrasound. Samples were treated with an Omni Ruptor 4000 sonicator provided by a titanium microtip of diameter 3.8 mm (OMNI International, the Homogenizer Company, Kennesaw, GA, USA). The sonication process was performed for 30, 20 and 10 min at a power of 210 W and a frequency of 20 kHz. To prevent excessive heating the samples were immediately placed in ice and water baths. Samples were stored at 4 °C. In the third method (SUAE), samples were shaken (200 rpm) at 28 °C for 1 or 7 days, and then subjected to ultrasound as before (power: 210 W, frequency: 20 kHz, Omni-Ruptor 4000, OMNI International Inc., USA with a Titanum 3/8″ Dia Solid tip).The obtained dry extracts were filtered using gravity filtration on a Whatman No. 4 filter (Millipore, USA) and then condensed under reduced pressure at 40 °C (Rotavapor R-215, Büchi, Switzerland). The condensed extracts were centrifuged (3900×*g*/10 min, centrifuge 5804R, Eppendorf, Poland) to eliminate wax depositing on the tube bottom. Next, the extracts were freeze dried (Alpha 1-4 LSC plus, Christ, Germany) and stored at 4 °C in dark containers (Bankova et al. 2016a; Graikou et al. 2016; Al-Ani et al. 2018; Al-Qurashi and Awad 2018; Escriche and Juan-Borrás 2018). The aforementioned procedures afforded a total of 12 EEPs (Table 1). Yields of all extraction processes were calculated by dividing the mass of the freeze dried extract by the total mass of raw propolis. Results were expressed in %. The percentage yields were calculated following Eq. (1):

**Table 1:** Extraction yield (%), total phenols and total flavonoids contents of the propolis extracts from shaking extraction (SE), ultrasonic-assisted extraction (UAE) and shaking with ultrasonic-assisted extraction (SUAE)

Trial abbreviation	Shaking time S (days)	Ratio of propolis to ethanol	Time of sonication U (min)	Extraction yield (%)	Total phenols (mg CAE/g)**	Total flavonoids (mg QE/g)***
SE						
S7-10-U0*	7	1:10	0	9.71 ± 0.76^d^	90.83 ± 6.27^c^	14.48 ± 0.04^c^
S7-5-U0	7	1:5	0	7.71 ± 0.65^e^	78.77 ± 6.73^d,e^	13.09 ± 0.07^e^
S1-10-U0	1	1:10	0	10.76 ± 0.84^c,d^	92.36 ± 2.34^b,c^	13.88 ± 0.15^d^
S1-5-U0	1	1:5	0	5.76 ± 0.51^f^	85.87 ± 2.71^c,d^	12.57 ± 0.03^f^
UAE						
S0-10-U10	0	1:10	10	8.35 ± 0.79^e^	76.03 ± 1.43^e^	11.01 ± 0.09^g^
S0-10-U20	0	1:10	20	10.73 ± 0.94^c,d^	81.76 ± 1.95^d^	13.12 ± 0.09^e^
S0-10-U30	0	1:10	30	11.86 ± 1.08^c^	98.74 ± 2.33^b^	15.07 ± 0.03^b^
S0-5-U20	0	1:5	20	10.08 ± 0.71^d^	89.61 ± 2.06^c,d^	13.75 ± 0.03^d^
SUAE						
S7-10-U20	7	1:10	20	15.92 ± 1.34^a^	105.29 ± 1.93^a^	15.69 ± 0.10^a^
S7-5-U20	7	1:5	20	11.64 ± 0.86^c^	94.64 ± 1.74^b,c^	15.71 ± 0.04^a^
S1-10-U20	1	1:10	20	14.19 ± 0.98^b^	104.16 ± 4.16^a^	15.23 ± 0.04^b^
S1- 5-U20	1	1:5	20	10.04 ± 0.88^d^	98.74 ± 2.95^b^	15.04 ± 0.06^b^

*S—shaking time (S7-7 days, S1-1 day, S0-without shaking), 5, 10—ratio of propolis to ethanol (5-ratio 1:5 and 10-ratio1:10), U—time of sonication (U30-30 min, U20-20 min, U10-10 min, U0-without sonication); **quercetin equivalent (QE)/g of propolis extract, ***caffeic acid equivalent (CAE)/g of propolis extract. a, b, c—mean values in the same column with different letters differ significantly (*p* ≤ 0.05)

1 $$ {\text{Yield}} = \left( {{\text{the}}\,{\text{ weight}}\,{\text{ of}}\,{\text{ the}}\,{\text{ extract}}\,{\text{ lyophilisate}}} \right)/\left( {{\text{the }}\,{\text{weight}}\,{\text{ of}}\,{\text{ crude}}\,{\text{ propolis}}} \right) \, \times \, 100 $$

### Determination of total flavonoid content

The total flavonoid content was measured by a modified method (Al-Ani et al. 2018). Initially, 4 mg of lyophilisate was dissolved in 10 mL of 50% ethanol. Briefly, 150 µL of EEP were mixed with 2% (w/w) AlCl_3_ (100 µL) in a 96-well microplate, then incubated at 37 °C for 30 min, and the absorbance at 415 nm was recorded with a Multiskan Sky Microplate Spectrophotometer (Thermo Fisher Scientific, USA) microplate reader against a blank (a sample without aluminum chloride). Quercetin was used as the standard. Quercetin standard solutions (0–10 mg/mL) were used for constructing the calibration curve (*y* = 0.3504*x* + 0.1514; *R*^2^ = 0.9936). The data were expressed as quercetin equivalent (QE) per g of propolis extract (Al-Ani et al. 2018).

### Determination of total phenolic content

The total phenolic content was measured by a modified method (Singleton et al. 1999). Briefly, 0.1 g of lyophilisate was dissolved in 10 mL of 50% ethanol. 15 µL of EEP sample extract and the standard solution (caffeic acid) with a concentration range of 0–200 µg/mL were pipetted into a round bottom 96-well plate. 240 µL of water and 15 µL of Folin–Ciocalteu solution were added to the well and left at 24 °C for 3 min. Then, 30 µL of 1.0 M Na_2_CO_3_ solution was added and mixed well. The plate was incubated at 24 °C for 2 h in the dark. The absorbances of the reactions were recorded at 765 nm with a Multiskan Sky Microplate Spectrophotometer (Thermo Fisher Scientific, USA) microplate reader against ethanol as a blank. Based on the measured absorbance of the caffeic acid (0–700 mg/mL), the calibration curve was constructed (*y* = 0.0009*x* + 0.0516; *R*^2^ = 0.9811). The contents of phenolic in the extracts were expressed in terms of caffeic acid equivalent (CAE). The total phenolic content was expressed in mg of caffeic acid equivalent (CAE)/g of extract (Singleton et al. 1999).

### Gas chromatography/mass spectrometry (GC/MS) analysis of propolis extracts

GC/MS was performed on a Shimadzu GCMS-QP2010S (Shimadzu, Japonia) equipped with a 30 m ZB-5 capillary column with a (5% phenyl)-polymethyl siloxane stationary phase, film thickness of 0.25 µm, diameter 0.25 mm. Briefly, 1 g of lyophilisate was dissolved in 10 mL of 50% ethanol. About 25–40 mg extract was evaporated under nitrogen conditions and derivatized with 50 µg pirydine, 100 µL BSTFA and 1% TMCS, and after 24 h was dissolved in 1 mL hexane. 1 µL of the sample was injected with a split mode (split ratio 1:25) with the carrier gas helium at a flow rate of 0.05 mL/min. The capillary column was coupled to a quadrupole mass spectrometer and the optimized instrumental parameters were as follows: Injector temperature (230 °C), head pressure (53.1 kPa), and transfer line heater (250 °C). The mass spectra were noted according to the following recommendations: Scan range (Routine): m/z 35–450, scan time: (Routine) 1 s, electron energy: 70 eV, source temperature: 220 °C, filament delay time: (Routine) 3 min; with GC MS Postrum Analysis (Kartal et al. 2002; Al-Ani et al. 2018).

### Determination of antimicrobial activity of ethanolic extracts of propolis

#### Test strains

Strains used in the tests included bacteria (*Staphylococcus aureus* ATCC 25923 and *Escherichia coli* O157 ATCC 700728) and fungi (*Candida krusei* ATCC 14243, *Mucor mucedo* ATCC 38694, *Alternaria solani* ATCC 16022, *Colletotrichum gloeosporioides* DSM 62146) obtained from the American Type Culture Collection or from the Leibniz Institute DSMZ-German Collection of Microorganisms and Cell Cultures., as well as moulds *Colletotrichum gloeosporioides* DSM 62146 obtained from the Leibniz Institute DSMZ-German Collection of Microorganisms and Cell Cultures. The strains were stored in 20% glycerol at − 80 °C in a freezer. The bacterial strains were cultured on Nutrient Agar (NA, BTL, Poland) at 37 °C for 24 h. Bacterial inocula were prepared in sterile saline (0.85% NaCl) (w/v) solution with the quantity corresponding to 0.5 McFarland (~ 1 × 10^8^ cfu/mL). Yeast was cultured on Sabouraud Dextrose Agar (SDA, BTL, Poland) at 28 °C for 48 h. The yeast inoculum was prepared in a sterile 0.85% NaCl (w/v) solution to reach a population of approximately ~ 1 × 10^6^ CFU/mL, using a hemacytometer. The mould conidia and spores were obtained from mycelium grown on SDA after incubation at 25 °C for 14 days. Spore or conidium suspensions were prepared in sterile 0.85% NaCl containing 0.1% Tween 80 to achieve ~ 1 × 10^6^ spores/mL, using a hemacytometer (Gniewosz et al. 2014).

#### Disc-diffusion method

Antimicrobial activities of EEP were determined with a disc-diffusion method (Standards Institute Clinical Laboratory (CLSI) 2006; Gavanji and Larki 2017). Briefly, 1 g of lyophilisate was dissolved in 10 mL of 50% ethanol. Sterile cellulose discs (6 mm diameter) were impregnated with 20 μL EEP (density 100 mg/mL). Equal EEP contents were obtained, and these were 2 mg d.w./disc. The suspensions of tested bacteria were spread evenly on the surface of MHA plates, and yeasts or mould spore suspensions on SDA plates. After 5 min, discs with EEP were placed on the surfaces of the inoculated plates. The plates with bacteria were incubated at 37 °C for 24 h, with yeast at 28 °C for 48 h, and those with moulds at 25 °C for 72 h. After incubation, the diameter of growth inhibition around the discs was measured. The results were expressed in millimeters. All tests were performed in triplicate and a new inoculum was prepared for each replicate, and the standard deviations were determined (Standards Institute Clinical Laboratory (CLSI) 2006; Gavanji and Larki 2017).

### Statistical analysis

Statistical tests were performed using the Statistica version 10PL computer program (StatSoft Inc., Poland). One-way analysis of variance was carried out. The significance of differences between mean values was assessed using the Tukey-test at a significance level of *α* = 0.05.

## Results

### Comparison of extraction yields

The yields of extractions varied between 5.76 and 15.92% depending on the method (Table 1). The yields of SE processes ranged from 5.76 ± 0.51 to 10.76 ± 0.84%. Statistically higher yields were observed for more diluted (1:10) as compared to less diluted (1:5) samples. The yields of UAE procedures increased with process duration and ranged from 8.35 ± 0.79% (S0-10-U10) to 11.86 ± 1.08% (S0-10-U30). The propolis to solvent ratio had no impact on the efficacy of extraction using this method. Extraction yields obtained using the SUAE method were higher than those obtained by either of the other methods. The highest yields of extraction were observed after 7 days of shaking extraction of samples diluted in a 1:10 ratio and subsequently subjected to UAE (S7-10-U20), as well as after 1 day of shaking extraction in otherwise similar conditions (S1-10-U20). Larger extraction yields were observed for the propolis to solvent ratio of 1:10 (compared to the 1:5 ratio).

### Comparison of total phenol and flavonoid content of EEPs

Table 1 presents total contents of polyphenols and flavonoids in ethanolic extracts of propolis obtained using the three extraction methods. The mean content of phenols following SE ranged from 78.77 mg CAE/g (S7-5-U0) to 90.83 mg CAE/g (S7-10-U0). The mean content of flavonoids in the same extracts ranged from 12.57 mg QE/g (S1-5-U0) to 14.48 mg QE/g (S7-10-U0). Overall, extracts obtained after 7 days of SE of samples with the propolis to solvent ratio of 1:10 were richer in flavonoids, whereas extracts obtained after 1 or 7 days of extraction of 1:10 samples were richer in phenols. For samples obtained using UAE, the content of phenols and flavonoids was found to increase along with the ultrasonication times. After 30 min of ultrasonication, the extract (S0-10-U30) with the highest polyphenol (98.74 mg CAE/g) and flavonoid content (15.07 mg QE/g) was obtained, whereas the extract subjected to only 10 min of ultraconication (S0-10-U10) presented with the lowest amounts of both classes of compounds: 76.03 mg CAE/g and 11.01 mg QE/g, respectively. Extracts obtained using the third method consisting in shaking extraction performed for 7 days and subsequent ultrasonic-assisted extraction lasting 20 min (S7-10-U20 and S7-5-U20) were found to contain statistically higher (*p* ≤ 0.05) quantities of flavonoids compared to the extracts obtained using the previously described methods. Statistically higher quantities of phenols (*p* ≤ 0.05) were observed for extracts S7-10-U20 (105.29 mg CAE/g) and S1-10-U20 (104.16 mg CAE/g). The content of flavonoids within the extracts obtained using the SUAE method ranged between 15.04 and 15.69 mg QE/g.

### Comparison of the content of identified components in individual EEPs

**Fig. 1:**
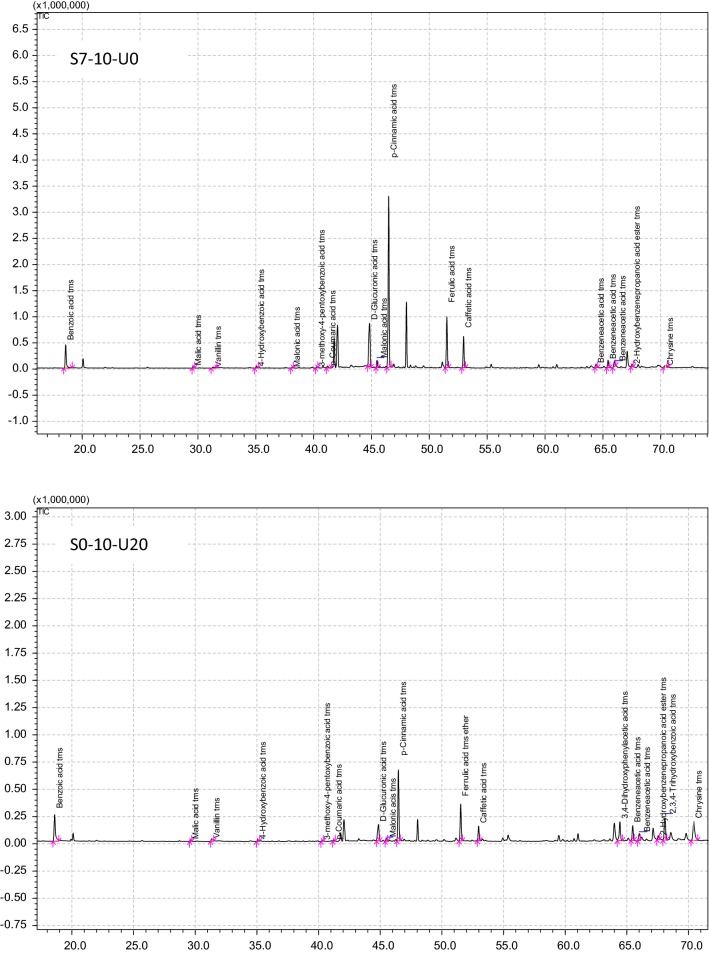

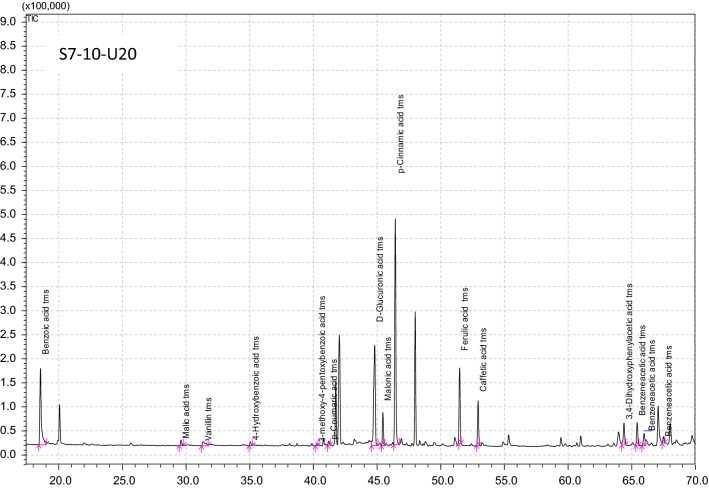
GLC/MS profil of chemical compounds of EEPs. *S—shaking time (S7-7 days, S0-without shaking), 10—ratio of propolis to ethanol (1:10), U—time of sonication (U20-20 min, U0-without sonication)

Three extracts, one per extraction method, were selected for the analysis of chemical composition using the GLC/MS technique. These included S7-10-U0, obtained following 7 days of shaking extraction, S0-10-U20 obtained after 20 min of ultrasound-assisted extraction, and S7-10-U20, obtained following 7 days of shaking extraction and 20 min of ultrasound-assisted extraction. Although the extracts differed in their total phenol and flavonoid contents, the components of these classes constituted the majority of all products. Figure 1 presents the chromatograms of chemical composition, while Table 2 compares the quantities of identified compounds in individual extracts. All selected extracts contained phenolic acids, including *p*-cinnamic acid, ferulic acid and caffeic acid, as well as carboxylic acids such as benzoic acid, benzeneacetic acid, 4-hydroxybenzoic acid, 3-methoxy-4-pentoxybenzoic acid, d-glucuronic acid, malic acid, and malonic acid. Likewise, all extracts contained acid derivatives such as 2-hydroxybenzenepropanoic acid ester and ferulic acid ether and four sugars, namely vanillin, maltose, sorbose and sucrose. An antibacterial flavonoid, chrysin, was present only in two of the extracts.

**Table 2:** Chemical components identified in propolis extracts by GLC/MS method

Chemical compound	Propolis extract		
	S7-10-U0^a^	S0-10-U20	S7-10-U20
Acetic acid tms	+	–	–
3,4-Dihydroxyphenylacetic acid tms	–	+	–
Benzeneacetic acid tms	+	+	+
Benzoic acid tms	+	+	+
2,3,4-Trihydroxybenzoic acid tms	–	+	–
3-Methoxy-4-pentoxybenzoic acid tms	+	+	+
4-Hydroxybenzoic acid tms	+	+	+
Butanoic acid tms	–	+	–
Caffeic acid tms	+	+	+
d-glucuronic acid tms	+	+	+
Ferulic acid	+	+	+
Hexadecanoic acid tms	+	–	–
Malic acid tms	+	+	+
Malonic acid tms	+	+	+
*p*-Cinnamic acid tms	+	+	+
2-Furanacetaldehyde tms	+	–	+
2-Hydroxybenzenepropanoic acid ester tms	+	+	+
Ferulic acid tms ether	+	+	+
Vanillin tms	+	+	+
d-xylose tms	–	+	–
Maltose tms	+	+	+
Mannose tms	+	+	–
Sorbose tms	+	+	+
Sucrose tms	+	+	+
Chrysine tms	+	+	–

^a^S—shaking time (S7-7 days, S0-without shaking), 10—ratio of propolis to ethanol (1:10), U—time of sonication (U20-20 min, U0-without sonication)

### Comparison of antimicrobial activities of EPPs

Table 3 presents the sizes of strain growth inhibition zones for extracts obtained using each of the three methods. All extracts were found to present with antimicrobial activity. The inhibitory activity of extracts varied not only depending on the extraction method, but also on the test species included in the assay. *S. aureus* was more sensitive to the study extracts than any other strain.

**Table 3:** Antimicrobial activity of propolis extracts from shaking extraction (SE), ultrasonic-assisted extraction (UAE) and shaking with ultrasonic-assisted extraction (SUAE)

Trial abbreviation	*S. aureus*	*E. coli*	*C. krusei*	*C. gloeosporioides*	*M. mucedo*	*A.solani*
	Zone of inhibition (mm ± SD**)					
SE						
S7-10-U0*	20.24 ± 0.76^a^***	17.28 ± 0.44^c^	13.89 ± 0.74^b,c,d^	16.61 ± 0.32^e,f,g^	12.64 ± 0.13^a^	9.66 ± 0.25^a^
S7-5-U0	24.45 ± 0.96^c^	14.73 ± 0.29^a^	15.27 ± 1.21^e,f^	16.08 ± 0.69^d,e,f^	13.41 ± 0.24^a,b^	13.01 ± 0.38^c^
S1-10-U0	25.38 ± 1.05^c,d^	15.72 ± 0.22^b^	13.45 ± 0.85^a,b^	14.62 ± 0.46^b,c,d^	14.62 ± 1.04^b,c,d^	15.37 ± 0.25^f^
S1-5-U0	22.43 ± 0.76^b^	15.88 ± 0.17^b^	13.67 ± 0.88^a,b,c^	14.03 ± 0.85^a,b,c^	13.46 ± 0.76^a,b^	11.48 ± 0.86^b^
UAE						
S0-10-U10	22.31 ± 0.82^b^	15.06 ± 0.41^a,b^	12.81 ± 0.41^a^	14.57 ± 0.21^b,c,d^	13.99 ± 0.58^a,b,c^	9.95 ± 0.50^a^
S0-10-U20	25.61 ± 0.98^c,d,e^	17.91 ± 0.61^c,d^	14.79 ± 0.84^d,e^	15.74 ± 1.16^c,d,e^	16.42 ± 0.25^e,f^	12.67 ± 0.60^b,c^
S0-10-U30	26.74 ± 0.74^e,f,g^	20.02 ± 0.50^f^	15.95 ± 0.73^f^	17.65 ± 0.22^f,g^	17.06 ± 0.17^g^	14.48 ± 1.00^d,e^
S0-5-U30	26.89 ± 0.67^f,g^	14.71 ± 0.27^a^	15.54 ± 0.69^e,f^	13.40 ± 0.36^a,b^	13.09 ± 1.05^a,b^	11.69 ± 0.10^b^
SUAE						
S7-10-U20	27.07 ± 0.87^f,g^	19.09 ± 0.32^d,e^	15.19 ± 0.43^e,f^	18.07 ± 0.61^h^	15.51 ± 1.10^d,e^	14.70 ± 0.66^d,e^
S7-5-U20	26.49 ± 1.73^d,e,f,g^	18.01 ± 0.15^c,d^	14.84 ± 0.55^d,e^	15.88 ± 0.90^c,d,e^	15.74 ± 0.26^d,e^	13.75 ± 0.40^c,d^
S1-10-U20	26.36 ± 0.54^d,e,f,g^	18.63 ± 0.30^d^	15.11 ± 0.86^e,f^	17.26 ± 0.72^f,g^	16.30 ± 0.72^e,f^	13.88 ± 0.36^c,d^
S1-5-U20	25.70 ± 0.79^d^	17.20 ± 0.39^c^	14.62 ± 0.82^c,d,e^	15.18 ± 0.70^c,d,e^	14.98 ± 0.89^c,d^	13.83 ± 0.65^c,d^

*S—shaking time (S7-7 days, S1-1 day, S0-without shaking), 5, 10—ratio of propolis to ethanol (5-ratio 1:5 and 10-ratio 1:10), U—time of sonication (U30-30 min, U20-20 min, U10-10 min, U0-without sonication), **Each value is the mean for three (*n* = 3) replicates, ***a, b, c—mean values in the same column with different letters differ significantly (*p* ≤ 0.05)

The sizes of strain growth inhibition zones for the propolis extract obtained using the shaking extraction technique ranged from 9.66 mm to 25.3 mm. Inhibition zones for *S. aureus* were larger (20.24–25.38 mm) than those for other microorganisms (9.66–17.28 mm). Higher variability of EEP activity was observed with regard to *S. aureus*, *C. gloeosporioides*, and *A. solani*, while low variability was observed with regard to *E. coli*, *C. krusei*, and *M. mucedo*. A significant impact of the propolis to solvent ratio was observed for the extract obtained in 7-day shaking extraction on the antimicrobial activity against all study strains. No such impact was observed for the extract obtained in 1 day shaking extraction with regard to *E. coli*, *C. krusei*, *C. gloeosporioides*, and *M. mucedo*, but not with regard to *S. aureus* and *A. solani*.

Samples obtained in ultrasound-assisted extraction were found to have antimicrobial activity similar to those obtained in shaking extraction. Most strain growth inhibition zone sizes ranged between 9.95 and 26.89 mm. The longer the time of ultrasonication, the higher the antimicrobial activity of the extracts. The largest strain growth inhibition zones were obtained in all tested strains for the extract subjected to 30 min of sonication (S0-10-30U). Samples subjected to 20 and 10 min of ultrasonication presented with lower strain growth inhibition activities. Reduction in the propolis to ethanol ratio from 1:10 to 1:5 led to reduced activity of the extract (S0-5-U20) against *E. coli*, *C. gloeosporioides*, and *M. mucedo* compared to the inhibitory activity of S0-10-U20.

Samples subjected to shaking extraction followed by ultrasound-assisted extraction (SUAE) were characterized by identical inhibitory activities against *S. aureus*, *E. coli*, *C. krusei*, and *M. mucedo*, with no statistical differences being observed in inhibition zone sizes (*p* > 0.05). The propolis to solvent ratio was found to affect the antifungal activity of extracts against *C. gloeosporioides* and *A. solani*, with growth inhibition zones being significantly larger (*p *≤ 0.05) for 1:10 extracts as compared to 1:5 extracts. No effect of shaking time on antifungal activity of extracts was observed.

## Discussion

In order to be suitable for use in the food industry, propolis extracts should present with high biological activity. Thus, an appropriate method for the preparation of crude propolis extracts is of key importance. In our study we compared the antimicrobial activity of propolis extracts obtained using three different methods: traditional shaking extraction (SE), ultrasound-assisted extraction (UAE), and shaking extraction combined with ultrasound-assisted extraction (SUAE). We observed that extracts obtained by shaking extraction combined with ultrasound-assisted extraction (SUAE) or ultrasound-assisted extraction (UAE) presented with higher antimicrobial activity than extracts obtained by traditional shaking extraction (SE) only. This was due to the higher overall extraction yield and higher contents of phenols and flavonoids in these extracts. Shaking extraction lasting 1 day followed by 20 min of ultrasound-assisted extraction (SUAE) increased the extraction of phenols by 15 and 23% and the extraction of flavonoids by 8 and 14% as compared to SE and UAE, respectively. The content of phenols and flavonoids determines the antimicrobial and antioxidative activity of propolis (Jug et al. 2014). Khacha-ananda et al. observed that the extraction of propolis by means of sonication techniques led to the total content of phenolic and flavonoid compounds being higher than in the case of maceration (Khacha-ananda et al. 2013). In other studies, the authors reported that UAE was superior to 2-day shaking extraction in terms of the extraction of flavonoids but inferior to the latter in terms of the extraction of phenolic compounds. Despite different content of bioactive compounds in the propolis extracts, no significant impact of these differences on antimicrobial properties was observed as all extracts inhibited the bacterial growth with similar strengths (Luján et al. 2018). Likewise, in the studies by Yeo et al. (2015), an extract with a considerably higher content of bioactive compounds obtained in an ultrasound-assisted procedure presented with a growth inhibition effect being only slightly higher for *Staphylococcus epidermidis* and *Bacillus subtilis* and lower for *E. coli* as compared to an extract obtained in a traditional, 1-day maceration process. This phenomenon may be explained by the complexity of composition of the propolis extracts and interactions between the effects of components which may be present even at very low levels (Bankova et al. 2016a).

The 1:10 versus 1:5 propolis to solvent ratio had no effect on the antimicrobial activity against most tested strains only for SUAE extracts. Extracts obtained by means of SE and UAE presented with lower inhibition of tested strains (*S. aureus*, *C. gloeosporioides*) when the propolis to solvent ratio was 1:5 than when the ratio was 1:10. The impact of the extraction mixture ethanol content on the quantities of bioactive agents within the final extract is difficult to explain as most researchers believe that the propolis to ethanol ratio has no impact on the extraction of most propolis components (Trusheva et al. 2007; Khacha-ananda et al. 2013).

Our results are in line with earlier studies which suggested that UAE significantly reduced the extraction times (Trusheva et al. 2007; Khacha-ananda et al. 2013; Jug et al. 2014). In addition, our studies showed that the longer the ultrasonication process, the stronger the inhibitory effect of extracts against test microorganisms. This phenomenon may be explained by the content of flavonoids and phenols being higher for longer sonication times.

Isidorov et al. (2014) investigated the chemical composition of various European propolis. In the Polish propolis, there are cinnamon acids and their derivatives, such as ferulic or coffeic acids. The content of cinnamon acids and their derivatives were estimated at 12%. Esters of cinnamon constituted 19.3% of the chemical composition of the extract. The content of chrysin was marked at 5.1%. The research on the chemical composition of Polish propolis was also carried out by Popova et al. (2017). Among the marked aromatic acids, the highest content was *p*-coumaric acid (5.3%) and ferulic acid (3.4%) and chrysin (3%) of all ingredients. Over a half of the chemical composition were esters of cinnamic acid and its derivatives. In ethanol extracts of propolis examined by Szliszka et al. (2013) also identified phenolic acids and their derivatives, and chrysin accounted for 6.56 mg/g of propolis.

The extraction method had no impact on the qualitative composition of propolis extracts. The antimicrobial properties of propolis are believed to be due to the presence of phenolic acids, including ferulic acid, cinnamic acid, benzoic acid, and benzeneacetic acid; these compounds were present in the tested extracts. We support the view that the biological activity of propolis is due to the synergistic action of all ingredients, not a specific chemical or group of ingredients (Boisard et al. 2015). Takasi et al. (1994) demonstrated that high levels of phenolic compounds lead to the denaturation of enzymes and consequently to bacterial cell death.

## Conclusion

The extraction method affects the antimicrobial properties of extracts, extraction yields, as well as the contents of phenolic and flavonoid compounds. Antimicrobial activity of extracts obtained by 1-day shaking extraction followed by ultrasonication was higher than extracts obtained by only traditional or ultrasound-assisted extraction. SE and UAE gave lower extraction yields as well as lower phenol and flavonoid contents compared to SUAE. SUAE gave a higher yield and higher content of phenols and flavonoids than SE and UEA.
